# A pilot study on acupuncture for lower urinary tract symptoms related to chronic prostatitis/chronic pelvic pain

**DOI:** 10.1186/1749-8546-2-1

**Published:** 2007-02-06

**Authors:** Jillian L Capodice, Zhezhen Jin, Debra L Bemis, David Samadi, Brian A Stone, Steven Kapan, Aaron E Katz

**Affiliations:** 1 Department of Urology, Columbia University Medical Center, New York, NY 10032, USA; 2 Department of Biostatistics, Columbia University Medical Center, New York, NY, 10032, USA; 3 Department of Urology, Weill Cornell Medical College, New York, NY, 10022, USA

## Abstract

**Background:**

The etiology and treatment of chronic prostatitis/chronic pelvic pain syndrome (CP/CPPS) remain poorly understood. Pain, lower urinary tract voiding symptoms and negative impact on quality of life (QOL) are the most common complaints. Acupuncture, which has been widely used to treat painful and chronic conditions, may be a potential treatment to alleviate the constellation of symptoms experienced by men with CP/CPPS. The purpose of our study was to assess the impact of standardized full body and auricular acupuncture in men refractory to conventional therapies and collect pilot data to warrant further randomized trials.

**Methods:**

Ten men diagnosed with category IIIA or IIIB CP/CPPS >6 months, refractory to at least 1 conventional therapy (antibiotics, anti-inflammatory agents, 5-α reductase inhibitors, α-1 blockers) and scoring >4 on the pain subset of the NIH-CPSI were prospectively analyzed in an Institutional Review Board (IRB) approved, single-center clinical trial (Columbia University Medical Center IRB#AAAA-7460). Standardized full body and auricular acupuncture treatment was given twice weekly for 6 weeks. The primary endpoints were total score of the NIH-CPSI and assessment of serious adverse events. The secondary endpoints were individual scores of the NIH-CPSI and QOL questionnaire scores of the short-form 36 (SF-36).

**Results:**

The median age of the subjects was 36 years (range 29–63). Decreases in total NIH-CPSI scores (mean ± SD) after 3 and 6 weeks from baseline (25.1 ± 6.6) were 17.6 ± 5.7 (P < 0.006) and 8.8 ± 6.2 (P < 0.006) respectively and remained significant after an additional 6 weeks of follow-up (P < 0.006). Symptom and QOL/NIH-CPSI sub-scores were also significant (P < 0.002 and P < 0.002 respectively). Significance in 6 of 8 categories of the SF-36 including bodily pain (P < 0.002) was achieved. One regression in the SF-36 vitality category was observed after follow-up. There were no adverse events.

**Conclusion:**

The preliminary findings, although limited, suggest the potential therapeutic role of acupuncture in the treatment of CP/CPPS. Data from this and previous studies warrant randomized trials of acupuncture for CP/CPPS and particular attention towards acupuncture point selection, treatment intervention, and durability of acupuncture.

## Background

The etiology for chronic prostatitis/chronic pelvic pain syndrome (CP/CPPS) has not been fully elucidated and the current treatment strategies for CP/CPPS are not universally accepted [1]. Chronic infection, inflammation, neuropathy, pelvic floor muscle dysfunction, autoimmune disease and neurobehavioral disorders are some of the postulated etiologies though no single factor is thought to be the absolute cause. Standard therapies for CP/CPPS include antibiotics, anti-inflammatory agents, 5-α reductase inhibitors, and α-1 blockers [1-3]. Recently, a multi-modal treatment approach and the utilization of complementary and alternative medicine (CAM) strategies such as acupuncture and phytotherapy have also been suggested as potential treatment options for CP/CPPS [4-7].

Acupuncture which has been used to treat painful and chronic conditions [8,9] may be useful in the treatment of pain, urinary and quality of life (QOL) symptoms frequently seen in men with CP/CPPS. While the etiology, treatment, long-term effects on QOL and CP/CPPS as a predictor of future prostate disease are yet to be determined, the rationale for safe and effective treatment practices for this complex condition is needed. This pilot study was implemented in order to test the efficacy of full body and auricular acupuncture on pain, urinary and QOL symptoms related to CP/CPPS and inform future research and randomized clinical trials of acupuncture for the management of symptoms related to CP/CPPS.

## Methods

### Patients

Our target sample size was 10 men diagnosed with CP/CPPS who were also refractory to conventional therapies. Subjects being treated at or referred to the Department of Urology, Columbia University Medical Center between April and September 2005 were offered participation in the study (Columbia University Medical Center, Institutional Review Board approved, IRB# AAAA-7460). The Department of Urology, Columbia University Medical Center, is located in New York (New York, USA) and has a large, diverse patient population. The inclusion criteria were men between the ages of 18 and 65, previous diagnosis with category IIIA or IIIB chronic prostatitis for >6 months, refractory to at least 1 conventional therapy (antibiotics, anti-inflammatory agents, 5-α reductase inhibitors or α-1 blockers), scoring >4 on the pain subset of the NIH-CPSI, and able to read and sign the informed consent.

Exclusion criteria were prostate cancer, bladder cancer, testicular cancer, stone disease, neurogenic bladder dysfunction, interstitial cystitis, urethritis, active sexually transmitted diseases (chlamydia, gonorrhea, syphilis, active herpes simplex virus) active urinary tract infection, bleeding disorders, skin infection, chronic use of anti-inflammatory agents (defined as use >2 times per week), active use of phytotherapeutic agents (saw palmetto, cernilton, quercetin) and previous acupuncture treatment within the past 6 months.

### Acupuncture intervention

**Figure 1:**
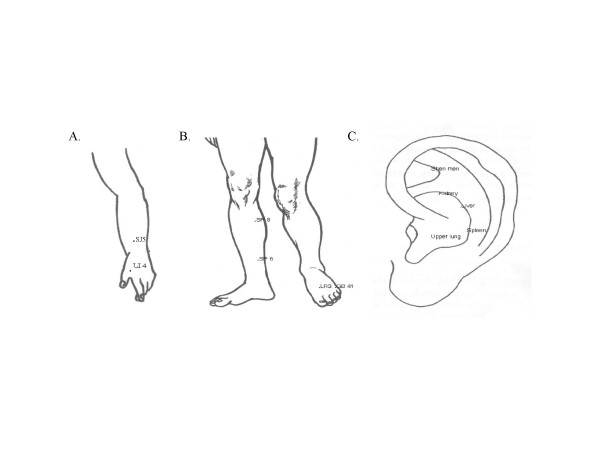
**Diagram of acupuncture points**. A. Acupuncture points located on the arm* B. Acupuncture points located on the legs* C. Auricular acupuncture points**. *These points were applied bilaterally. **These points were applied in one ear and alternated with each treatment visit.

The protocol consisted of a standardized set of acupuncture points given twice weekly for 30 minutes over 6 weeks based on traditional Chinese medicine organ (*zang-fu*) and meridian theory. Points were selected based on standard texts, informal practitioner query and expertise, and selected points from previous studies [6,10-12]. Standardized full body and auricular acupuncture was performed by JC throughout the study. An initial informal piloting of the protocol included enactment of the standardized point prescription in two adult, consenting and non-study patients. Acupuncture needles were single-use, sterile and disposable. Full body acupuncture needles were 25 mm or 40 mm (34 gauge) and auricular needles were 15 mm (36 gauge). The needling protocol consisted of first needling auricular points, followed by full-body points. Needles were inserted to the proper needling depth as determined by standard point locations [10-12] and a *de qi *sensation was obtained. *De qi *is defined as a feeling of soreness, numbness, distention, or heaviness around the point after the needle is inserted to a certain depth, meanwhile, the practitioner may feel a sense of tenseness around the needle [10]. The needles remained *in situ *for 20–25 minutes during which time the acupuncturist returned to stimulate the needles once utilizing scraping (defined as scraping the handle of the needle up to six times) and even needle technique (defined as lightly rotating the needle back and forth up to six times) in order to re-elicit the *de qi *sensation. The body acupuncture points given at each visit included SJ 5-*wai guan *(alternate name TB 5), GB 41-*zulin qi*, LR 3-*tai chong*, LI 4-*he gu*, SP 8-*di ji *and SP 6-*san yin jiao*. The auricular acupuncture points needled at each visit in one ear and alternating ears with each treatment included *shen men*, kidney, liver, lung and spleen (Figure 1). There were no additional co-interventions.

### Outcome measures

#### NIH-CPSI

The subjects enrolled in the study completed the standardized National Institute of Health chronic prostatitis symptom index (NIH-CPSI) at baseline (0 week), midpoint (3 weeks), endpoint (6 weeks) and follow-up (12 weeks).

#### Short-form 36 (SF-36)

The subjects enrolled in the study completed the standardized short-form 36 (SF-36) at baseline (0 week), midpoint (3 weeks), endpoint (6 weeks) and follow-up (12 weeks).

All investigators except ZJ (statistician) were blinded to the questionnaire answers and study results until completion of the study.

### Sample size and statistical analysis

We determined our sample size based on previous studies [6,7] and determination of treatment effect with a maximum allowable difference of 0.1 and confidence level of 95% of our primary outcome measure, i.e. the NIH-CPSI.

Each of the measures was summarized by mean ± standard deviation at each measurement week (0, 3, 6, 12). The changes of symptom measures at weeks 3, 6 and 12 compared to baseline (0 week) were assessed by the paired Wilcoxon signed test. P-values less than 0.05 are considered significant. All statistical analyses were carried out with the statistical software package SAS version 9.1.

## Results

Of 14 men screened for the study, 10 fit the inclusion/exclusion criteria and were enrolled into the study following informed consent. The median age of the 10 subjects was 36 years (range 29 to 63). The previous use of conventional therapies, i.e. antibiotics, non-steroidal anti-inflammatory drugs, 5-α reductase inhibitors and α-1 blockers, was as follows: antibiotics (quinilones: levofloxacin or ciprofloxacin) 10 of 10, non-steroidal anti-inflammatory drugs (ibuprofen, naproxen, cyclooxygenase-2 inhibitors) 6 of 10, and 5-α reductase inhibitors (finasteride) 3 of 10, and α-1 blockers (terazosin) 1 of 10. All 10 patients completed the study period and assessments at the scheduled time except for the 6-week follow-up (median 6 weeks, range 5.5–7.1).

The decreases in the NIH-CPSI scores (mean ± SD) after 3 weeks (17.6 ± 5.7), 6 weeks of acupuncture (8.8 ± 6.2) and 6 weeks of follow-up (6.6 ± 4.3) from baseline (25.1 ± 6.6) were remarkably significant (P < 0.006). Decreases in the total symptom (pain + urinary) and quality of life (QOL) components of the NIH-CPSI were also considered significant (P < 0.002 and P < 0.002 respectively) (Table 1). The changes of QOL measures utilizing the SF-36 at weeks 3, 6 and 12 compared to baseline confirmed significant changes in 6 of 8 categories of the SF-36 after 6 weeks of acupuncture treatment (Table 2). The significant categories included physical function (P < 0.016), role limitations due to physical health (P < 0.016), role limitations due to emotional health (P < 0.047), vitality (P < 0.037), social functioning (P < 0.008) and bodily pain (P < 0.002). One regression in the vitality category was observed after the 12 week follow-up (P = 0.111) (Table 2). There were no reported adverse events or important medical events as defined by our data and safety-monitoring plan. One case of slight bruising at GB 41 was reported in a single patient on one occasion. No other side effects were reported.

**Table 1:** Mean (SD) summary statistics of the NIH-CPSI score in 10 patients.

**NIH-CPSI**	**Baseline 0**	**wk 3**	**P values** ** 3 wk vs. 0**	**wk 6**	**P values** ** 6 wk vs. 0**	**wk 12**	**P values ** **12 wk vs. 0**
Total score	25.1 (6.6)	17.6 (5.7)	0.006*	8.8 (6.2)	0.006*	6.6 (4.3)	0.006*
Pain scores	9.9 (3.1)	5.3 (4.2)	0.004*	3.1 (2.0)	0.002*	2.1 (2.0)	0.002*
Urinary score	5.7 (3.3)	5.6 (2.5)	0.984	1.7 (2.5)	0.004*	1.6 (1.2)	0.008*
QOL score	9.5 (2.0)	6.7 (2.7)	0.004*	4.1 *2.6)	0.002*	2.9 (2.0)	0.002*
Symptom score = Pain + Urinary	15.6 (5.2)	10.9 (4.1)	0.002*	4.8 (4.0)	0.002*	3.7 (2.9)	0.002*

CPSI: National institutes of health – chronic prostatitis symptom index

* significant: exact Wilcoxon Signed Test P-values

**Table 2:** Mean (SD) summary statistics of SF-36 QOL in 10 patients.

**SF-36 Subsets**	**Baseline 0**	**wk 3**	**wk 6**	**wk 12**	**3 wk vs. 0**	**6 wk vs. 0**	**12 wk vs. 0**
Physical function	83(15.5)	96(9.4)	97.5(4.2)	98.5(3.3)	0.070	0.016*	0.023*
Role limitation due to physical health	10(10.7)	18.1(10.4)	22.5(7.3)	21.3(7.9)	0.133	0.016*	0.063
Role limitation due to emotional problems	10.8(11.1)	19.2(7.9)	20.8(8.1)	22.5(4.0)	0.148	0.047*	0.027*
Vitality	38.1(11.2)	48.1(13.2)	55.6(16.0)	50.6(17.8)	0.035*	0.037*	0.111
Mental health	63.5(17.0)	64.0(20.1)	72.0(10.9)	76.0(6.6)	0.844	0.211	0.066
Social functioning	43.8(8.8)	52.5(9.9)	57.5(8.7)	60.0(7.9)	0.063	0.008*	0.008*
Bodily pain	45.1(16.1)	76.0(20.1)	87.3(8.8)	88.8(16.5)	0.006*	0.002*	0.004*
General health	47.0(16.0)	58.0(22.5)	60.0(16.0)	61.5(12.3)	0.234	0.061	0.086

SF-36: Short-form 36

*significant: exact Wilcoxon Signed Test P-values

## Discussion

Though the etiology of CP/CPPS remains poorly understood, the gold standard of treatment is the use of fluroquinilones as first-line therapy [1-3]. Meanwhile, the application of self-reported NIH-CPSI implemented in 1999 by the Chronic Prostatitis Collaborative Research Network (CPCRN) is useful in determining the primary subjective patient symptoms [13-15]. This questionnaire has demonstrated a capacity in capturing the qualitative symptoms of men with CP/CPPS [15].

### NIH-CPSI

The data in our study demonstrate the safety of acupuncture and a significant treatment effect on the overall and individual pain, urinary and QOL symptoms as measured by administration of the NIH-CPSI following acupuncture treatment (2 times per week over 6 weeks). While absolute efficacy endpoints can not be established based on our uncontrolled study, trends regarding specific acupuncture application and treatment effects related to duration of treatment were noted in our study. The significant reduction in pain sub-scores of the NIH-CPSI following 3 weeks of treatment versus significant reduction in urinary sub-scores only after 6 weeks of treatment implies that the treatment effect of acupuncture for pain is quicker acting than it may be for urinary symptoms. The effect of acupuncture on pain was further supported by decreased scores in the bodily pain category of the SF-36, which were also significant after 3 weeks of treatment.

### Quality of life

Emotional health and QOL with regard to social and sexual functioning has been observed in past studies of chronic prostatitis and co-morbid depression has also been postulated as a potential factor in the etiology of CP/CPPS [16,17]. Interestingly, while none of our study patients were on any current anti-depressant medications, further analysis of the SF-36 revealed a significant change in role limitations due to emotional problems versus a non-significant change in mental health sub-component (following 3 and 6 weeks of treatment). Although a full mental health profile was not assessed in our study, these results are consistent with the hypothesis that chronic prostatitis may cause significant social and sexual distress but may not be related to co-morbid depression [18,19].

### Previous studies

Further analysis of our study versus two previous pilot studies testing acupuncture for CP/CPPS also needs to be addressed [6,7]. It is curious to note that all three studies (ours included) used three different acupuncture point prescriptions and protocols (Table 3) and these data demonstrate the complexity of acupuncture clinical trial design including research methodology challenges, such as the selection of appropriate point prescription, needling techniques and standardized protocols.

**Table 3:** Brief characteristics of the three acupuncture studies for CP/CPPS.

**Author**	**Sample size**	**CP/CPPS**	**Acupuncture points**	**Duration**	**Significance**
Chen *et al*. 2003	n = 12	IIIA/B	Set 1: BL10,23,28,40, KI 10 K1, BL67-Bilateral Set 2: CV3, 4, M-CA-17, SP 6 + 5 hz e-stim Set 3: B:23, BL54, BL35	Three sets of points given alternately twice/week over 6 weeks. 20 minute treatment*	yes CPSI
Honjo *et al*. 2004	n = 10	IIIB	BL 33-Bilateral	Single point applied bilaterally over 4 weeks. 10 minute treatment*	yes CPSI and IPSS
Capodice *et al*. 2007	n = 10	IIIA/B	GB 41, LR 3, LI 4, SJ 5, SP 8, SP 6-Bilateral *Shen men*, kidney, liver, lung, spleen and one auricle	Twice/week full body and auricular acupuncture over 6 weeks. 20–25 minute treatment (needles *in situ*)+	yes CPSI

CPSI: National institutes of health – chronic prostatitis symptom index

IPSS: International Prostate Symptom Scores

* no detail on needling procedure

+ detail on needling procedure includes attainment of *de qi *and needle technique

Both of the previous studies reported significant effects of acupuncture utilizing the NIH-CPSI, whereas the selections of acupuncture points were different between them without authors' further explaining of point selection rationales [6,7]. By contrast, our study had a clear aim to assess a standardized full body and auricular acupuncture point prescription based on traditional Chinese medicine organ (*zang-fu*) and meridian theory. Our points were selected in compliance with the traditional principles of moving *qi*, easing blood stagnation, relieving pain, and opening up the meridians (Table 4). In addition, all three studies used the same endpoint, namely scores on the NIH-CPSI, and all three studies obtained significance after six weeks of treatment [6,7].

**Table 4:** Traditional functions of acupuncture points*.

**Name**	**Functions/Point Category**
*San Jiao *5 (Triple Burner 5) – *Wai guan*	Frees the channels and quickens the connecting vessels, clears heat and resolves toxin, dissipates wind; *Luo *point of the *san jiao *channel, confluence point of the 8 extraordinary channels
Gall Bladder 41-*Zulin qi*	Courses liver and gallbladder *qi *stagnation, clears fire and extinguishes wind, transforms obstructing phlegm-heat; *Shu *point, confluence point of the 8 extraordinary channels
Liver 3-*Tai chong*	Extinguishes liver fire, clears liver yang, discharges damp-heat in the lower burner, soothes liver *qi*, frees the channels and quickens the connecting vessels; *Shu *point, *yuan *source point
Large Intestine 4-*He gu*	Frees the channels and quickens the connecting vessels, relieves pain and quiets the spirit; *Yuan *source point
Spleen 8-*Di ji*	Harmonizes the spleen and rectifies blood; *Xi*-cleft point
Spleen 6-*San yin jiao*	Supplements spleen, helps movement and transformation, frees *qi *stagnation, courses the lower burner; Intersection point of spleen channel, one of nine needles for returning yang
Auricular-*shen men*	Promotes relaxation, relieves pain
Auricular-kidney	Strengthens the kidneys
Auricular-liver	Strengthens gastrointestinal tract, relieves muscle spasms
Auricular-lung	Relieves pain
Auricular-spleen	Strengthens the gastrointestinal tract, strengthens following a disease

*Adapted from references [10] and [12]

While these data suggest that acupuncture is beneficial for symptoms related to CP/CPPS, a number of research methodological issues, to be addressed in future studies, were present in the current study. These include selection of appropriate and homogenous treatment groups with regard to CP/CPPS such as duration of condition (e.g. >6 months to upwards of 20 years), previous and current biomedical treatment(s) and refractory status. Issues concerning acupuncture include high expectation of acupuncture for refractory patients, utilization of an appropriate, inert sham-control, acupuncture point selection and application of points within or independent of a traditional medicine framework.

### Potential physiologic mechanisms of action

Research on the hypothesized mechanism of action of acupuncture for CP/CPPS reveals challenges in both CP/CPPS research and acupuncture research. As aforementioned, the exact etiology of CP/CPPS is not understood and no single treatment strategy has been deemed effective. Moreover, the exact mechanism of action of acupuncture is not clear. Regarding the use of acupuncture for CP/CPPS, the mechanism of action suggested in one of the previous studies implies a neuropathic etiology of CP/CPPS and suggests that one of the treatment effects of acupuncture is of a neuro-physiological nature [6].

While we agree that the hypothesis of a neuropathic model seems plausible, elucidating the exact physiological mechanisms of acupuncture for CP/CPPS may ultimately equally demand determination of the exact pathology of CP/CPPS including the discovery of new surrogate endpoints, biomarkers, and neuromodulatory mediators involved in inflammation, endocrine function and their implication in CP/CPPS pathology. We also hypothesize that acupuncture may have a multi-modal treatment effect(s) which may include pain relief via potential anti-inflammatory and neuromodulatory mechanisms as well as positive impact on QOL potentially involving non-specific effects of acupuncture such as patient-practitioner interaction. These new theories begin to introduce a biopsychosocial model and mechanism of CP/CPPS in diagnostic and treatment strategies and may be useful in future CP/CPPS studies of acupuncture and other new treatments [20-25].

### Limitations

Limitations of this study include small sample size, non-randomized, non-sham controlled trial design, possible placebo effect and investigators' bias that may exaggerate the findings of the study.

In addition, acupuncture was tested in a group of refractory patients. A recent study testing the use of ciprofloxacin, tamsulosin or both in refractory CP/CPPS men showed no clinical effect in any category, and concern regarding investigation of agents in previous treatment failures is notable [26]. Recently, the Chronic Prostatitis Collaborative Research Network [26] and Habmermacher *et al*. [27] have proposed a treatment algorithm for newly diagnosed CP/CPPS patients dependent on subjective symptoms and a few clinical trials. This algorithm suggests the application of first to third-line medications and therapies depending on most acute symptoms and historical treatment outcomes for each related symptom [27]. Therefore, the study of acupuncture as a potential first-line therapy or as an adjuvant therapy depending on primary patient who reported subjective symptoms may be of interest in our future studies. In addition, the complexity and controversy surrounding the design of an appropriate placebo for acupuncture research need to be addressed. Taken together, these challenges facing both conventional and non-conventional treatment options for CP/CPPS may generate greater understanding and a successful treatment for men with CP/CPPS.

## Conclusion

The preliminary findings of this pilot study suggest a significant and safe treatment effect by acupuncture as measured by a validated questionnaire, for pain and lower urinary tract symptoms experienced by men with CP/CPPS. Data from this and previous studies warrant randomized trials of acupuncture for CP/CPPS in order to investigate the mechanism of action of acupuncture in CP/CPPS, via rigorous clinical trial design that may begin to address the complexity of acupuncture research including rationales for acupuncture point selection, treatment duration, and the ability to determine the effectiveness and durability of acupuncture in larger, randomized and multi-center trials.

## Abbreviations

CAM: Complementary and alternative medicine

CP/CPPS: Chronic prostatitis/chronic pelvic pain syndrome

NIH-CPSI: National Institute of Health chronic prostatitis symptom index

SD: Standard deviation

SF-36: Short-form 36

QOL: Quality of life

## Competing interests

The author(s) declare that they have no competing interests.

## Authors' contributions

JLC was responsible for study design, acupuncture treatment, manuscript preparation and submission. ZJ was responsible for all statistical design and analysis of data. DLB was responsible for manuscript review. BAS was responsible for patient recruitment. DS was responsible for patient recruitment. SK was responsible for manuscript review and patient recruitment. AEK was responsible for study design, critical manuscript review and patient recruitment. All authors read and approved the final manuscript.

## References

[B1] Kreiger JN, Ross SO, Penson DF, Riley DE (2002). Symptoms and inflammation in chronic prostatitis/chronic pelvic pain syndrome. Urology.

[B2] Nickel JC (2004). The three As of chronic prostatitis therapy: antibiotics, alpha-blockers and anti-inflammatories. What is the evidence?. BJU Int.

[B3] Pontari MA, Ruggieri MR (2004). Mechanisms in prostatitis/chronic pelvic pain syndrome. J Urol.

[B4] Shoskes DA (2002). Phytotherapy and other alternative forms of care for the patient with prostatitis. Curr Urol Rep.

[B5] Potts JM (2005). Therapeutic options for chronic prostatitis/chronic pelvic pain syndrome. Curr Urol Rep.

[B6] Chen R, Nickel JC (2003). Acupuncture ameliorates symptoms in men with chronic prostatitis/chronic pelvic pain syndrome. Urology.

[B7] Honjo H, Kamoi K, Naya Y, Ukimura O, Kojima M, Kitakoji H, Miki T (2004). Effects of acupuncture for chronic pelvic pain syndrome with intrapelvic venous congestion: preliminary results. Int J Urol.

[B8] Audette JF, Ryan AH (2004). The role of acupuncture in pain management. Phys Med Rehabil Clin N Am.

[B9] National Center for Complementary and Alternative Medicine website. http://nccam.nih.gov.

[B10] Chen X, (ed) (1999). Chinese Acupuncture and Moxibustion.

[B11] Wu Y, Fisher W (1997). Practical Therapeutics of Traditional Chinese Medicine.

[B12] Huang LC (1997). Auriculotherapy diagnosis and treatment.

[B13] Litwin MS (2002). A review of the development and validation of the National Institutes of Health Chronic Prostatitis Symptom Index. Urology.

[B14] Litwin MS, McNaughton-Collins M, Fowler FJ, Nickel JC, Calhoun EA, Pontari MA, Alexander RB, Farrar JT, O'Leary MP (1999). The National Institutes of Health chronic prostatitis symptom index: Development and validation of a new outcome measure. J Urol.

[B15] Propert KJ, Alexander RB, Nickel JC, Kusek JW, Litwin MS, Landis JR, Nyberg LM, Schaeffer AJ, Chronic Prostatitis Collaborative Research Network (2002). Design of a multicenter randomized clinical trial for chronic prostatitis/chronic pelvic pain syndrome. Urology.

[B16] Casey J (2006). Changes in symptoms and quality of life in patients with chronic prostatitis. Nat Clin Pract Urol.

[B17] Propert KJ, McNaughton-Collins M, Leiby BE, O'Leary MP, Kusek JW, Litwin MS, Chronic Prostatitis Collaborative Research Network (2006). A prospective study of symptoms and quality of life in men with chronic prostatitis/chronic pelvic pain syndrome: the National Institutes of Health Chronic Prostatitis Cohort study. J Urol.

[B18] Ku JH, Kim SW, Paick JS (2005). Quality of life and psychological factors in chronic prostatitis/chronic pelvic pain syndrome. Urology.

[B19] Tripp DA, Curtis Nickel J, Landis JR, Wang YL, Knauss JS, CPCRN Study Group (2004). Predictors of quality of life and pain in chronic prostatitis/chronic pelvic pain syndrome: findings from the National Institutes of Health Chronic Prostatitis Cohort Study. BJU Int.

[B20] Coeytaux RR, Kaufman JS, Kaptchuk TJ, Chen W, Miller WC, Callahan LF, Mann JD (2005). A randomized, controlled trial of acupuncture for chronic daily headache. Headache.

[B21] David J, Townsend S, Sathanathan R, Kriss S, Dore CJ (1999). The effect of acupuncture on patients with rheumatoid arthritis: a randomized, placebo-controlled cross-over study. Rheumatology.

[B22] Chen Y, Song B, Jin XY, Xiong EQ, Zhang JH (2005). Possible mechanism of referred pain in the perineum and pelvis associated with the prostate in rats. J Urol.

[B23] Shahed AR, Shoskes DA (2001). Correlation of beta-endorphin and prostaglandin E2 levels in prostatic fluid of patients with chronic prostatitis with diagnosis and treatment response. J Urol.

[B24] Nadler RB, Koch AE, Calhoun EA, Campbell PL, Pruden DL, Bennett CL, Yarnold PR, Schaeffer AJ (2000). IL-1beta and TNF-alpha in prostatic secretions are indicators in the evaluation of men with chronic prostatitis. J Urol.

[B25] Zijlstra FJ, van den Berg-de Lange I, Huygen FJ, Klein J (2003). Anti-inflammatory actions of acupuncture. Mediators Inflamm.

[B26] Alexander RB, Propert KJ, Schaeffer AJ, Landis JR, Nickel JC, O'Leary MP, Pontari MA, McNaughton-Collins M, Shoskes DA, Comiter CV, Datta NS, Fowler JE, Nadler RB, Zeitlin SI, Knauss JS, Wang Y, Kusek JW, Nyberg LM, Litwin MS, Chronic Prostatitis Collaborative Research Network (2004). Ciprofloxacin or tamsulosin in men with chronic prostatitis/chronic pelvic pain syndrome: a randomized, double-blind trial. Ann Intern Med.

[B27] Habermacher GM, Chason JT, Schaeffer AJ (2006). Prostatitis/Chronic Pelvic Pain Syndrome. Annu Rev Med.

